# Nanofibrous Photocatalytic Membranes Based on Tailored Anisotropic Gold/Ceria Nanoparticles

**DOI:** 10.1021/acsami.1c11954

**Published:** 2021-07-30

**Authors:** Yinzhou Guo, Héloïse Thérien-Aubin

**Affiliations:** † Max Planck Institute for Polymer Research, Mainz 55128, Germany; ‡ Department of Chemistry, Memorial University of Newfoundland, St. John’s, Newfoundland and Labrador A1B 3X7, Canada

**Keywords:** anisotropic nanoparticle, gold, CeO_2_, colloidal electrospinning, nanofibers, photocatalysis

## Abstract

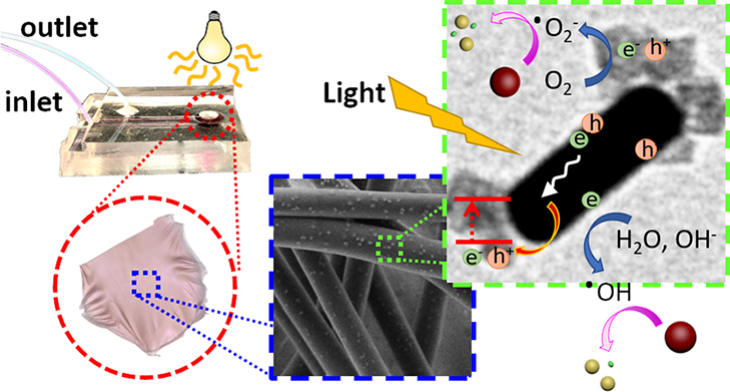

The combination of plasmonic nanoparticles with semiconductor photocatalysts is a good strategy for synthesizing highly efficient photocatalysts. Such binary nanoparticles have demonstrated enhanced catalytic activity in comparison to either plasmonic catalysts or semiconductor catalysts. However, problematic recovery and limited long-term colloidal stability of those nanoparticles in suspension limit their wide use in catalysis. To palliate to such limitations, we embedded binary nanoparticles in polymer fibers to design photocatalytic membranes. First, we used the selective over-growth of crystalline cerium oxide on the gold nanoparticle template with distinct shapes. Gold nanospheres, gold nanorods, and gold nanotriangles were used as the template for the growth of the cerium oxide domains. Then, the resulting nanoparticles were used to catalyze model reactions in suspensions. The gold nanoparticles covered with patches of cerium oxide outperformed the fully covered and naked nanoparticles in terms of catalytic efficiency. Finally, the most efficient binary nanostructures were successfully embedded in nanofibrous membranes by colloidal electrospinning and used in water remediation experiments in a flow-through reactor.

## Introduction

Among the array of photocatalysts currently being developed, plasmonic-driven and plasmonic-enhanced nanoparticle (NP) photocatalysts are particularly appealing. They can generate hot charge carriers produced by local surface plasmon resonance, which are typically more energetic than the electrons generated by traditional semiconductors and organic photocatalysts.^1^ Consequently, such NPs can carry out photocatalytic reactions under very mild reaction conditions.^2^ However, the practical application of plasmonic photocatalysts NPs in suspensions remains restricted, being hampered by a lack of long-term colloidal stability and recyclability. To circumvent those limitations, the immobilization of NPs in a solid matrix, like electrospun fibers, is appealing. The optimization of the plasmon-enhanced photocatalytic NPs followed by their embedding in nanofibrous mats can yield a highly efficient filtration membrane compatible with a continuous process like their implementation in flow-through reactors.

An extensive range of plasmonic metal NPs (including Au and Ag) has been used as photocatalysts^3,4^ mainly because they can generate hot carriers following the optical excitation of their localized surface plasmon resonances.^5−7^ One of the main advantages of plasmonic photocatalyst NPs is their unmatched ability to combine light absorption, electric field, energetic carriers, and thermal effect in one material. The hot charge carriers, produced by the interaction with light, can migrate to the surface of the NP where they can activate the chemical transformations of reactants located in close proximity to the surface. The efficiency of the ensuing chemical reactions is influenced by both the interaction between the reactants and the NPs and the efficiency of the transfer of the hot charge carriers from the NPs to the reactant.^6−9^ Consequently, the size and shape of the NPs can influence the catalytic activity. For example, small AuNPs have higher surface-to-volume ratios; this allows for the more efficient transfer of the hot carriers to the surface of the particle. Consequently, small AuNPs exhibit a higher photocatalytic activity than larger AuNPs. Furthermore, the shapes of the particle influence the absorption of light and thus the formation of hot charge carriers. Therefore, understanding the effect of the NP size and shape on the catalytic activity would provide guidelines for designing photocatalytic materials.

The combination of plasmonic NPs with semiconductors can largely improve the photocatalytic efficiency of the NPs.^10−12^ The functionalization of plasmonic NPs with semiconductor domains creates a new generation of highly efficient catalysts for visible-light-driven photocatalysis, and the resulting metal–semiconductor hybrid NPs display potential for applications in a large scope of fields such as dye-sensitive solar cells (DSSCs), photocatalysis, photoelectric devices, or hydrogen production.^13−16^ Different metal NPs (including Au, Pt, and Ag) have been combined with various semiconductors, such as TiO_2_, ZnO, and CdS.^16−19^ The properties of metal–semiconductor NPs are highly dependent on the size, shape, and spatial distribution of each component, but the resulting hybrid NPs are more photocatalytically efficient than either pure plasmonic NPs, pure semiconductor NPs, or a mixture of the two individual NPs.^16^

Among all the semiconductor NPs used for catalysis, cerium oxide displays a high oxygen storage capacity, a large number of oxygen vacancy defects between the oxidation states of cerium(III) and cerium(IV), and good thermal stability behavior.^20^ These characteristics lead to enhanced reaction rates and make CeO_2_ a prime candidate for the design of metal–semiconductor hybrid NPs.^21^ The combination of CeO_2_ with AuNPs is particularly appealing because CeO_2_ has an appropriate Schottky barrier. The energy of the Schottky barrier of CeO_2_ is similar to the energy of the hot electrons generated by the plasmonic AuNPs, facilitating their injection in the CeO_2_ domains,^22−25^ making the CeO_2_-AuNP hybrid nanomaterials ideal for plasmonic-enhanced photocatalysis.

Even though metal–semiconductor binary NPs are very efficient photocatalysts, their direct applications remain difficult due to their challenging long-term colloidal stability, demanding separation from the reaction medium, and overall problematic recovery and recycling. Embedding the NPs in a solid matrix is a convenient way to facilitate their straightforward applications. For example, porous solids, clays, and microbeads have been used among other substrates to immobilize photocatalytic NPs.^26−28^ An alternative approach is the immobilization of the NPs in or on nanofibers prepared by colloidal electrospinning.^29,30^

Electrospinning is a scalable and versatile technique to obtain nanofibrous materials with variable porous structures and controllable fiber diameters.^31^ Electrospinning is a process where an electric field is used to produce fibers, often using polymers. In this process, a polymer solution or melt is pumped through an electrified capillary nozzle. The applied high voltage generates charges at the surface of the liquid. The difference of potential also accelerates the liquid toward a target. The combined effects of the electrical field on the liquid lead to the elongation of the fluid and the formation of a jet, which is then transformed either by the drying or phase transition into fibers. The morphology and size of the resulting fibers can be tuned by the different parameters of the electrospinning process. These include the potential applied during the spinning process, the solvent or solvent mixture used, the distance between the nozzle and the target, or the presence of additives. Electrospinning of polymers and polymer hybrids has already been implemented in roll-to-roll processes and other industrial processes to form self-standing fibers, mats, and coatings.^32−35^

During colloidal electrospinning, preformed NPs can be combined to polymer solutions or melts to produce hybrid nanofibers.^36−39^ Furthermore, the immobilization of photocatalytic NPs within electrospun fibrous mats leads to the formation of catalytic membranes,^40^ which are more convenient for handling, processing, and avoiding contamination of the catalytic systems.^41^ Recently, hybrid electrospun mats containing a variety of NPs have found application in a range of fields such as wound dressing materials and antifouling coatings.^42,43^ However, the efficiency of the photocatalytic electrospun fibers can be improved by refining the design of the photocatalytic NPs embedded in the fibers, which would pave the way to using the resulting membranes in a continuous process.

**Figure 1:**
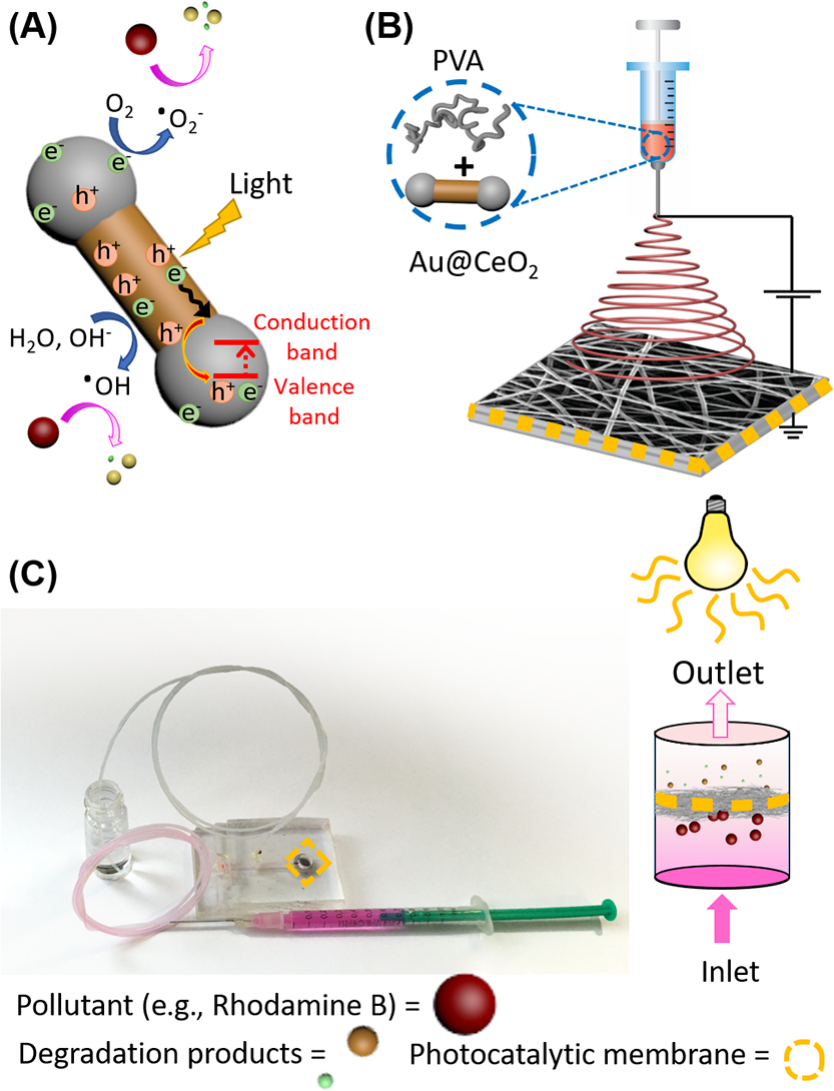
Preparation of photocatalytic membranes by the colloidal electrospinning of suspension of poly(vinyl alcohol) (PVA) and gold-cerium oxide nanoparticles and their use in a flow-through fluidic device. (A) Photocatalysis on Au@CeO_2_ nanoparticles, (B) production of photocatalytic nanofibrous Au@CeO_2_/poly(vinyl alcohol) hybrid membranes by colloidal electrospinning, and (C) photocatalysis with the flow-through reactor containing a nanofibrous hybrid membrane.

Herein, we designed self-standing photocatalytic fibrous polymer mats containing AuNPs functionalized with CeO_2_ domains. We used the over-growth of CeO_2_ on specifically shaped AuNP templates to prepare a library of tunable binary AuNP@CeO_2_ catalysts. By turning the structure of the AuNP templates and the distribution of CeO_2_ domains, we could control the photocatalytic activity of the resulting binary NPs. The AuNP@CeO_2_ binary NPs can promote both benchmark reduction and oxidation reaction of model waste products used in water remediation. This present approach combines the advantage of a metal NP and semiconductor patches, providing a platform for the design of novel hybrid metal–semiconductor anisotropic particles, which can be used as building blocks for the development of functional mesoscopic structures. To demonstrate this potential, we embedded the most efficient binary NPs in poly(vinyl alcohol) (PVA) fibers and used the resulting nanofibrous mats to perform photocatalytic reactions in a continuous process (Figure 1).

## Experimental Section

### Materials

Hydrogen tetrachloroaurate(III) (HAuCl_4_) (40% solution in dilute hydrochloric acid), sodium hydroxide (NaOH), phenol red, sodium borohydride (NaBH_4_), ascorbic acid (AA), silver nitrate, potassium iodide (KI), potassium platinum(II) chloride, cetyltrimethylammonium bromide (CTAB), and cetyltrimethylammonium chloride (CTAC) were purchased from Sigma-Aldrich; hydrogen peroxide solution (30%) and cerium acetate (Ce(AC_3_)) were purchased from Roth; poly(vinyl alcohol) (PVA) (*M*_w_ = 125,000 g·mol^–1^, 88% hydrolyzed) was purchased from Polysciences Inc., and glutaraldehyde (GA, 50% aqueous solution) was purchased from Merck. All experiments performed in an aqueous environment were conducted using Milli-Q water (pH = 6.90, 18.2 MΩ).

### Synthesis of Gold Nanospheres (Au NSs)

Au NSs were synthesized using a modified seed-mediated growth method.^44^ The seed solution was prepared by adding 0.6 mL of a freshly prepared ice-cold solution of NaBH_4_ (10 mM) to 9.833 mL of aqueous CTAB (0.1 M) and then mixed with 0.167 mL of HAuCl_4_ (15 mM) in a 100 mL Erlenmeyer flask. After vigorous stirring for 2 min, the pale yellow mixture was left undisturbed for 40 min at 25 °C and then diluted to 100 mL with the addition of Milli-Q water. Next, the growth solution was prepared by mixing 4 mL of CTAB solution (0.24 M), 0.133 mL of HAuCl_4_ (15 mM), and 3 mL of ascorbic acid solution (0.1 M). The growth solution was subsequently diluted to 50 mL with Milli-Q water. Then, 0.6 mL of the seed solution was added to the growth medium. The mixture solution was kept in a water bath at 30 °C for 24 h. After the reaction, the excess of CTAB and unreacted species were removed by two cycles of centrifugation (20,000*g*, 20 min at 25 °C) followed by redispersion in CTAB solution (0.1 mM).

### Synthesis of Gold Nanorods (Au NRs)

Au NRs were synthesized using a seed-mediated growth method.^45^ The seed solution was prepared by adding 0.6 mL of freshly prepared ice-cold NaBH_4_ solution (10 mM) to a mixture containing 0.12 mL of HAuCl_4_ (15 mM) and 2.5 mL of CTAB solution (0.2 M). The seed solution was kept at 25 °C for 40 min before use. Next, the growth solution was prepared by mixing 5 mL of CTAB solution (0.2 M) with 0.5 mL of HAuCl_4_ solution (15 mM), 0.4 mL of AgNO_3_ solution (4 mM), and 4 mL of Milli-Q water. The dark yellow growth solution turned colorless after the addition of 0.124 mL of ascorbic acid solution (0.0788 M). Then, 0.1 mL of the seed solution was added to the growth solution, and the mixture was reacted in a water bath at 30 °C for 24 h without stirring. After the reaction, the excess of CTAB and unreacted salts were removed by two cycles of centrifugation (18,000*g*, 20 min at 25 °C) followed by redispersion in CTAB solution (0.1 mM).

### Synthesis of Gold Nanotriangles (Au NTs)

Au NTs were synthesized using a seedless procedure.^46^ First, a mixture was prepared by combining 8 mL of Milli-Q water, 1.6 mL of CTAC solution (0.1 M), 75 μL of KI solution (0.01 M), 80 μL of HAuCl_4_ solution (25.4 mM), and 20.3 μL of NaOH solution (0.1 M) in a 50 mL Erlenmeyer flask. Once the mixture became homogeneous, 80 μL of ascorbic acid (0.064 M) was added to the reaction. Then, the solution became colorless after 30 s of moderate shaking, and 10 μL of NaOH (0.1 M) was then added quickly under ultrasonication for 5 s. The color of the solution changed from colorless to blue within ca. 20 min. After the reaction, the excess of CTAC and unreacted salts were removed by three cycles of centrifugation (10,000*g*, 20 min at 25 °C) followed by redispersion in Milli-Q water for the first two cycles and redispersion in CTAB solution (0.1 mM) after the third centrifugation cycle.

### Synthesis of the Binary Nanoparticles AuNP@CeO_2_

Following the synthesis of the AuNP templates, binary NPs were prepared by the selective adsorption of PtCl_4_^–^ followed by the oxidation of Ce(AC)_3_ at high temperature (Figure S1). Typically, 1 mL of the as-prepared AuNP suspension was centrifuged and redispersed with 0.5 mL of CTAB solution (0.1 mM). Then, different amounts of K_2_PtCl_4_ solution (0.1 mM) were added to the AuNP suspension under gentle shaking (see the Supporting Information for details). The resultant suspension was kept at room temperature for 30 min to allow for the adsorption of PtCl_4_^2–^ on AuNPs. Then, a freshly prepared Ce(AC)_3_ solution (10 mM) and water were added to the suspension. Finally, the resultant suspension was placed in an oven set at 100 °C for 1 h to produce the AuNP@CeO_2_ binary NPs. The unreacted salts were removed by two centrifugation cycles followed by redispersion in water.

### Photocurrent Measurement

Suspensions of AuNPs, either AuNSs, AuNTs, or AuNRs, or their binary NPs prepared with CeO_2_ were dried on 0.25 cm^2^ poly(ethylene terephthalate) (PET) film coated with indium tin oxide (ITO). The amount of gold deposited on the PET film was kept constant to 2 mg. The current density measurements were carried out using a three-electrode system. PET/ITO coated with NPs was used as the working electrode, in conjunction with a Ag/AgCl electrode in saturated KCl as the reference electrode and a platinum wire as the counter electrode. The electrodes were immersed in a solution of NaOH (1 M). The measurements were performed with a Methrom Autolab PGSTAT 204 system controlling the illumination provided by an air-cooled LED (OLM-018, OSA Opto Light GmbH).

### Characterization of the Catalytic Activity in Solution

#### Catalytic Reaction of 4-Nitrophenol (4-NP)

First, 25 μL of the 4-NP aqueous solution (either 10, 15, or 25 mM) was combined to 3 mL of a freshly prepared aqueous solution of NaBH_4_ (8 mg·mL^–1^). At this stage, 4-NP was converted to a nitrophenolate anion. Then, the appropriate volume (10, 30, and 50 μL) of Au@CeO_2_ suspension containing 0.2 mg·mL^–1^ gold was added. The mixture was transferred into a quartz cuvette and irradiated with white light using an air-cooled LED (OLM-018, OSA Opto Light GmbH) with an optical power density of 2 W·cm^–2^. UV–vis absorption spectrometry was used to monitor the conversion after a predetermined reaction time.

#### Catalytic Reaction of Phenol Red

First, an aqueous solution containing NH_4_Br (20 mg·mL^–1^), H_2_O_2_ (200 mg·mL^–1^), and phenol red (0.25 mg·mL^–1^) was prepared. Then, the appropriate volume (150, 250, and 350 μL) of Au@CeO_2_ suspension containing 0.2 mg·mL^–1^ gold was added to 2.5 mL of this phenol red solution. The mixture was transferred into a quartz cuvette and irradiated with white light using an air-cooled LED (OLM-018, OSA Opto Light GmbH) with an optical power density of 2 W·cm^–2^. UV–vis absorption spectrometry was used to monitor the conversion after a predetermined reaction time.

#### Catalytic Reaction of Rhodamine B

An aqueous solution of rhodamine B (0.0015 mg·mL^–1^) was prepared. Then, 50, 100, and 150 μL of the Au@CeO_2_ suspension containing 0.2 mg·mL^–1^ gold were added to 2.5 mL of this rhodamine B solution. The mixture was transferred into a quartz cuvette and irradiated with white light using an air-cooled LED (OLM-018, OSA Opto Light GmbH) with an optical power density of 2 W·cm^–2^. UV–vis absorption spectrometry was used to monitor the conversion after a predetermined reaction time.

### Electrospinning and PVA/AuNR@CeO_2_ Composite Nanofibers

Suspensions of AuNR with different coverages of CeO_2_ were prepared with a constant concentration of gold (60 mg·mL^–1^). Then, those concentrated AuNR@CeO_2_ suspensions (1 mL) were combined with a PVA solution (2 mL, 0.15 g·mL^–1^) to yield a final suspension containing 0.1 g·mL^–1^ PVA and 20 mg·mL^–1^ Au. The PVA/AuNR suspensions were ultrasonicated for 10 min and then stirred overnight at 200 rpm to obtain homogeneous suspensions. For electrospinning (Figure 1), a 1 mL syringe was filled with the PVA/AuNR mixture, and electrospinning was conducted at room temperature with a relative humidity of 20–25%. The fibers were collected onto an aluminum foil carrier placed at a distance of 20 cm from the nozzle (diameter, 0.8 mm). The flow rate was 0.3 mL·h^–1^, and the applied voltage was +18 kV. Fibers without any AuNRs were prepared under the same conditions. Samples for transmission electron microscopy (TEM) analysis were prepared by placing a TEM grid (copper, 300 mesh) on the aluminum foil to collect the fibers for 15 s.

The fibers were cross-linked (Figure S2) by placing fiber mats in a reaction chamber with a vial containing 1 mL of a 50 wt % GA solution and 20 μL of a 37 wt % HCl solution. The pressure in the reaction chamber was reduced to 1 mbar, and the reaction was carried out at room temperature for 24 h. The reaction chamber was vented and the mats recovered. Finally, any unreacted GA and HCl remaining in the mats were removed by drying the samples for 24 h at room temperature in the fume hood.

### Preparation of the In-Flow Reactor

**Figure 2:**
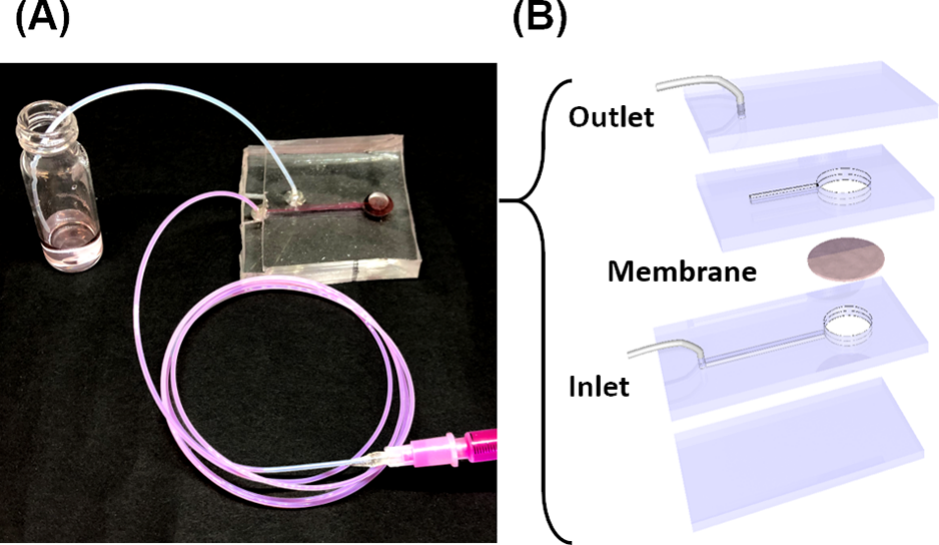
Architecture of the flow-through device. (A) Photograph of the device. (B) Structure of the different PDMS layers building the device.

Polydimethylsiloxane (PDMS) layers were prepared by curing Sylgard 184 on a bas-relief of the channels at 70 °C overnight. The outlets and the reaction chamber were created in the PDMS layers with a punch borer. Coupons of the electrospun membrane (ø = 1 cm) were cut with a punch borer. PDMS was treated in an oxygen plasma chamber for 30 s to promote the bonding between the PDMS layers, and then the membrane coupon was sandwiched between the flat side of two PDMS layers patterned with the channels and the reaction chambers (Figure 2). The resulting stack was then bonded with two unpatterned PDMS layers to close the channels. Finally, tubings (PTFE, i.d. 1/32) were fixed at the inlet and outlet holes with epoxy glue. The devices were then kept at 70 °C before use, at least for 4 h.

### Catalytic Activity of the Electrospun Membranes

An aqueous solution of rhodamine B (0.0015 mg·mL^–1^) was injected into the inlet of the device prepared with different membranes using a syringe pump (Harvard Instrument, PHD Ultra) at a flow rate varying from 0.5 to 5 mL·h^–1^. The solution was collected at the outlet and analyzed by UV–vis spectroscopy to measure the conversion defined as the difference in the concentration of rhodamine B between the inlet and the outlet normalized by the initial concentration of rhodamine B. The light source (LED (OLM-018, OSA Opto Light GmbH)) was positioned 5 cm above the device.

## Results and Discussion

### Synthesis of the Au@CeO_2_ Nanoparticles

Gold nanoparticles (AuNPs), either gold nanospheres (AuNSs), gold nanorods (AuNRs), or gold nanotriangles (AuNTs), were selectively functionalized with a complete or partial layer of cerium oxide (CeO_2_) through an over-growth process to yield binary AuNP@CeO_2_ (Figure S1). The site over-growth of CeO_2_ on the anisotropic AuNPs occurs through the use of PtCl_4_^2–^ ions as the guiding units. The PtCl_4_^2–^ ions bind more strongly to high index facets of the AuNPs^24^ and are the locus of the site-specific oxidation of the ceria precursor. When the system was fed with a limiting amount of K_2_PtCl_4_, the platinum ions would preferentially adsorb on those high index facets. Then, the ceria precursor, like cerium acetate (Ce(AC)_3_), can be rapidly hydrolyzed into Ce(OH)_3_ when the temperature is higher than 60 °C and subsequently be oxidized by the preadsorbed PtCl_4_^2–^ through an autoredox reaction to produce CeO_2_ nuclei in the locations where PtCl_4_^2–^ ions were present. Further growth of those CeO_2_ nuclei yields localized patches of CeO_2_ when an excess of the ceria precursor is heated above a temperature of 100 °C. Such an approach was used to generate patchy anisotropic binary AuNP@CeO_2_.

The amount of PtCl_4_^2–^ used played an essential role in the site-selective growth of CeO_2_, given that the over-growth of CeO_2_ only happens at the gold surface area where PtCl_4_^2–^ ions were adsorbed. Using AuNRs as the starting material, either fully covered NRs (AuNR@F-CeO_2_) were produced when an excess of K_2_PtCl_4_ was used or tip-covered NRs (AuNR@T-CeO_2_) were produced in the presence of a limiting amount of K_2_PtCl_4_. Similarly, fully covered AuNTs (AuNR@F-CeO_2_), edge-covered AuNTs (AuNT@E-CeO_2_), or tip-covered AuNTs (AuNR@T-CeO_2_) can be produced by limiting the amount of K_2_PtCl_4_ used. When AuNSs were used as the substrate, a limiting concentration of K_2_PtCl_4_ during the reaction yielded Janus NPs (AuNS@J-CeO_2_) while an excess of K_2_PtCl_4_ yielded fully covered nanostructures (AuNS@F-CeO_2_).

**Figure 3:**
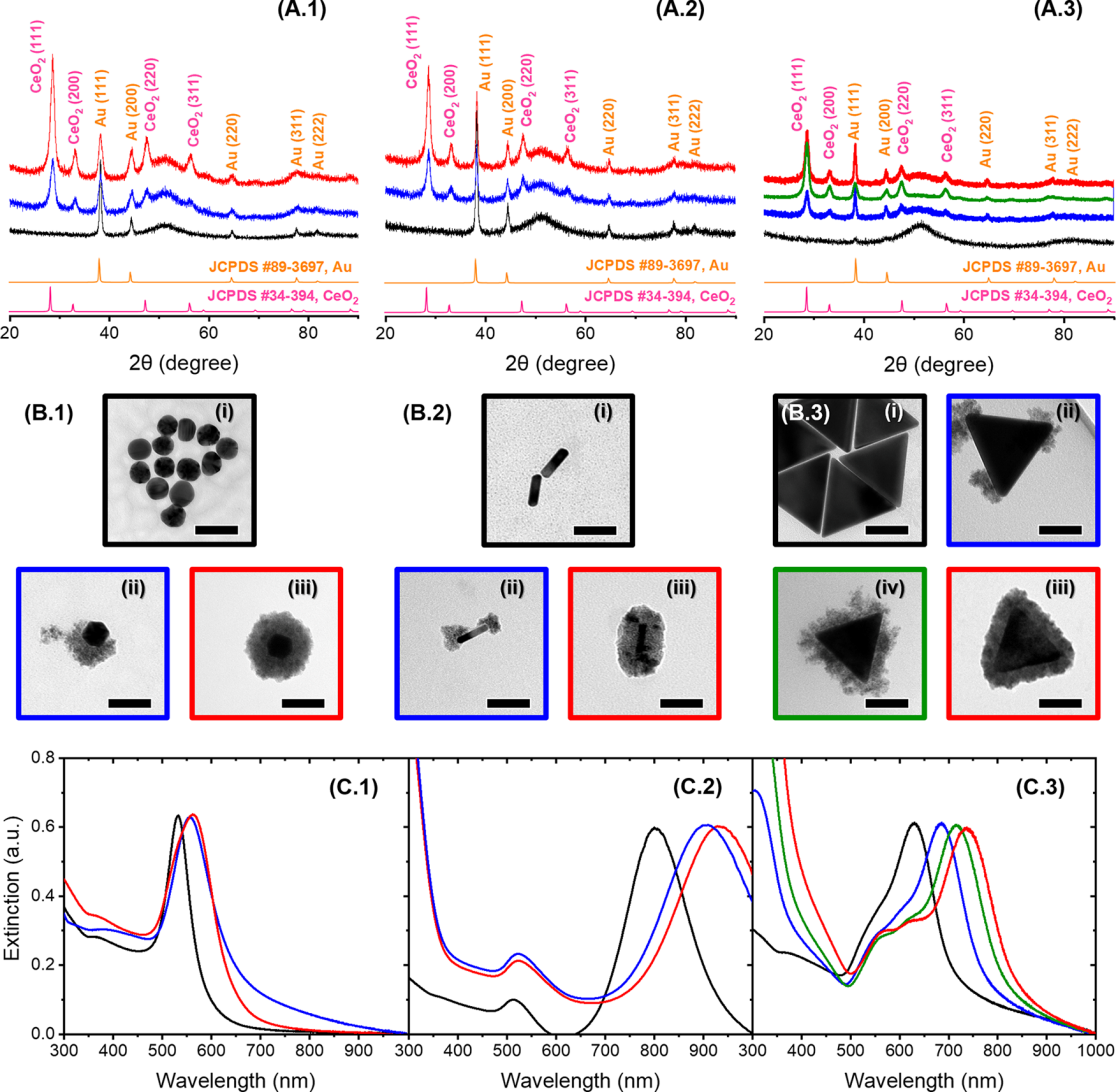
Characterization of the binary nanoparticles prepared with templates of (1) AuNS, (2) AuNR, and (3) AuNT. For nanoparticles that are either (i) naked NPs (black), (ii) Janus or tip-covered NPs (AuNS@J-CeO_2_ or AuNP@T-CeO_2_) (blue), (iii) fully covered NPs (AuNP@F-CeO_2_) (red), or (iv) edge-covered NPs (AuNT@E-CeO_2_) (green). (A) XRD patterns of the binary NPs and the reference diffraction patterns for Au (JCPDS #89-3697) (orange) and CeO_2_ (JCPDS #34-394) (pink). (B) TEM images of the resulting NPs. The scale bars are 50 nm. (C) UV–vis extinction spectra of AuNP@CeO_2_ binary patchy nanoparticles.

X-ray diffraction (XRD) of the binary NPs (Figure 3A) showed two sets of diffraction peaks, one stemming from the AuNP and one ascribed to the CeO_2_ over-growth. Similar results were obtained for the CeO_2_-modified AuNS, AuNR, and AuNT. Therefore, one set of diffraction peaks was assigned to the cubic phase of CeO_2_ (JCPDS #34-394) and the other one to the cubic phase of Au (JCPDS #89-3697).

The resulting NPs were analyzed by transmission electron microscopy and UV–vis spectroscopy (Figure 3B and Figure 3C, respectively). The results show that the modification of the AuNSs with CeO_2_ over-growth led to a red-shift in the localized surface plasmon resonance (LSPR) extinction. The addition of CeO_2_ onto the naked AuNS resulted in the red-shift of the LSPR maxima, which was more pronounced for the fully covered AuNS@CeO_2_ than the partially covered AuNS@CeO_2_. This shift was due to an increase in the local refractive index of the immediate surrounding of the AuNSs, and the increase in the average local refractive index was more significant as the amount of CeO_2_ on the AuNS increased. A larger fraction of the surface area of the AuNS covered with CeO_2_ resulted in a larger increase in the average local refractive index.^47^ Similar results were also observed with the AuNRs and AuNTs, with the main difference being the intrinsic extinction of the NPs being influenced by the shape of the NPs leading to different types of plasmon excitation modes.^48−51^

### Photocatalysis in Suspension

The excitation of LSPR of AuNPs generates “hot electrons” that possess high kinetic energy after being accelerated by the strong electric field of the plasmon. Those hot electrons can be harnessed to catalyze an array of organic reactions involving species either in close vicinity or adsorbed to the gold surface.^52,53^ The catalytic activity of the AuNP@CeO_2_ binary nanostructures was investigated using three different benchmark reactions: the reduction reaction of 4-nitrophenol to 4-aminophenol in the presence of sodium borohydride, the oxidation reaction of phenol red to bromophenol blue in the presence of hydrogen peroxide and halogen ions, and the photodegradation of rhodamine B.^54−56^ Those reactions are model reactions used in the development of photocatalytic water remediation strategies.^57,58^

**Figure 4:**
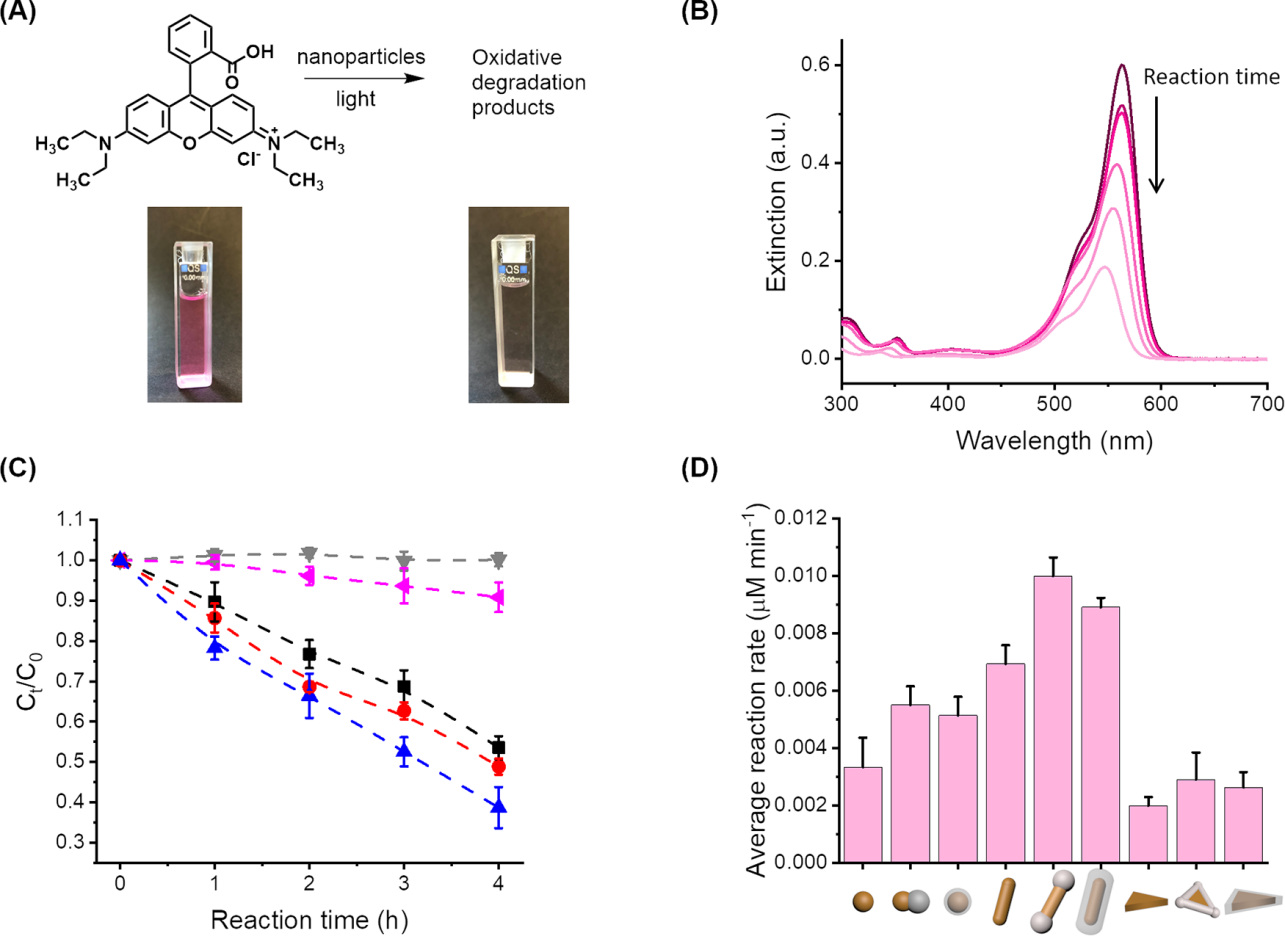
Catalytic activity of the nanoparticles in suspension during the photooxidation of rhodamine B. (A) Degradation of rhodamine B. (B) UV–vis spectra of the catalytic degradation of rhodamine B. (C) Variation of the concentration of rhodamine B during the reaction in the presence of AuNRs or AuNR@CeO_2_ under different conditions. Light irradiation, without AuNRs (upside down triangle, grey); with AuNRs, without irradiation (left-pointing triangle, pink); with AuNRs, under irradiation (square, black); with AuNRs@T-CeO_2_, under irradiation (triangle, blue); and with AuNRs@F-CeO_2_, under irradiation (circle, red). (D) Reaction rate of the photodegradation of rhodamine B in the presence of the different AuNP@CeO_2_.

Rhodamine B can be degraded under light irradiation in the presence of photocatalysts like AuNPs or AuNP@CeO_2_. During rhodamine B degradation, the absorption peak at ca. 554 nm decreased, and the color of the solution changed from pink to colorless (Figure 4A). This reaction was used as a benchmark to compare the library of AuNP@CeO_2_ binary NPs prepared by monitoring the concentration of rhodamine B in solution by UV–vis spectrometry (Figure 4B). Similarly, the oxidation of phenol red to bromophenol blue in the presence of Br^–^ and H_2_O_2_ can be catalyzed with light after the addition of the right NPs. During this reaction, the color of the solution turned from light yellow (phenol red) to blue (bromophenol blue), and consequently, the absorption peak at ca. 400 nm belonging to phenol red decreased, while the peak belonging to bromophenol blue at ca. 590 nm increased. The reaction kinetics could be monitored from time-dependent UV–vis spectrometry (Figure S3). Likewise, the reduction of 4-nitrophenol (4-NP) to 4-aminophenol (4-AP) in the presence of NaBH_4_ was accompanied by a decrease in intensity of the peak at ca. 400 nm in the UV–vis spectra of 4-NP (Figure S4) while the 4-AP peak at ca. 300 nm increased and can be used to quantify the extent of the reaction (Figure S4).

**Figure 5:**
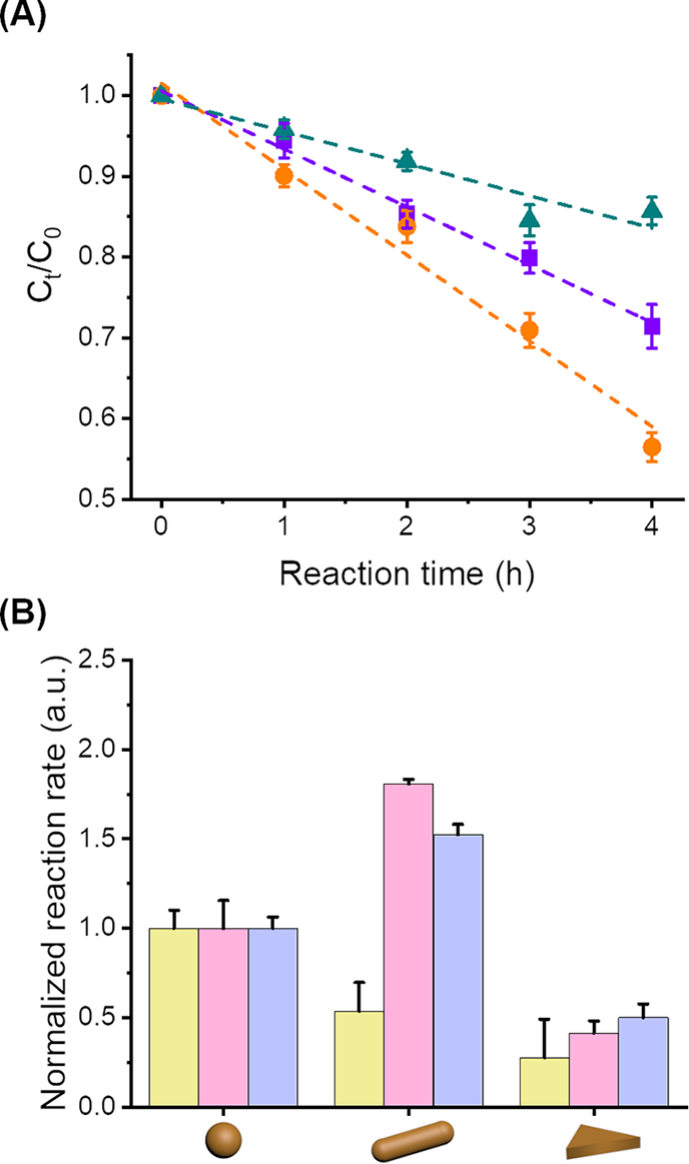
(A) Variation of the concentration of rhodamine B during photodegradation catalyzed with AuNS (square, purple), AuNR (circle, orange), and AuNT (triangle, cyan). (B) Normalized reaction rate for the catalytical reaction of 4-nitrophenol to 4-aminophenol (yellow), of phenol red to bromophenol blue (blue), and the degradation of rhodamine B (pink) in the presence of naked gold nanoparticles of different shapes.

To compare the reaction rate observed in the presence of NPs with different shapes and architectures, all the reactions were performed at a constant concentration of gold in suspension at 0.2 mg·mL^–1^. First, the effect of the shape was analyzed (Figure 5). In every case, in the presence of NPs but in the absence of light, the reagents (either rhodamine B, phenol red, or 4-NP) were not consumed; similar results were also observed for reagent solution irradiated in the absence of NPs. However, when the NPs were present in suspensions and irradiated with white light, the reagents were consumed, as measured by UV–vis spectrometry (Figure 5A). The photocatalytic activity of the AuNPs resulted from plasmon excitation, which is shape-specific, and the different AuNP templates used affected the catalytic activity observed (Figure 5B).

**Figure 6:**
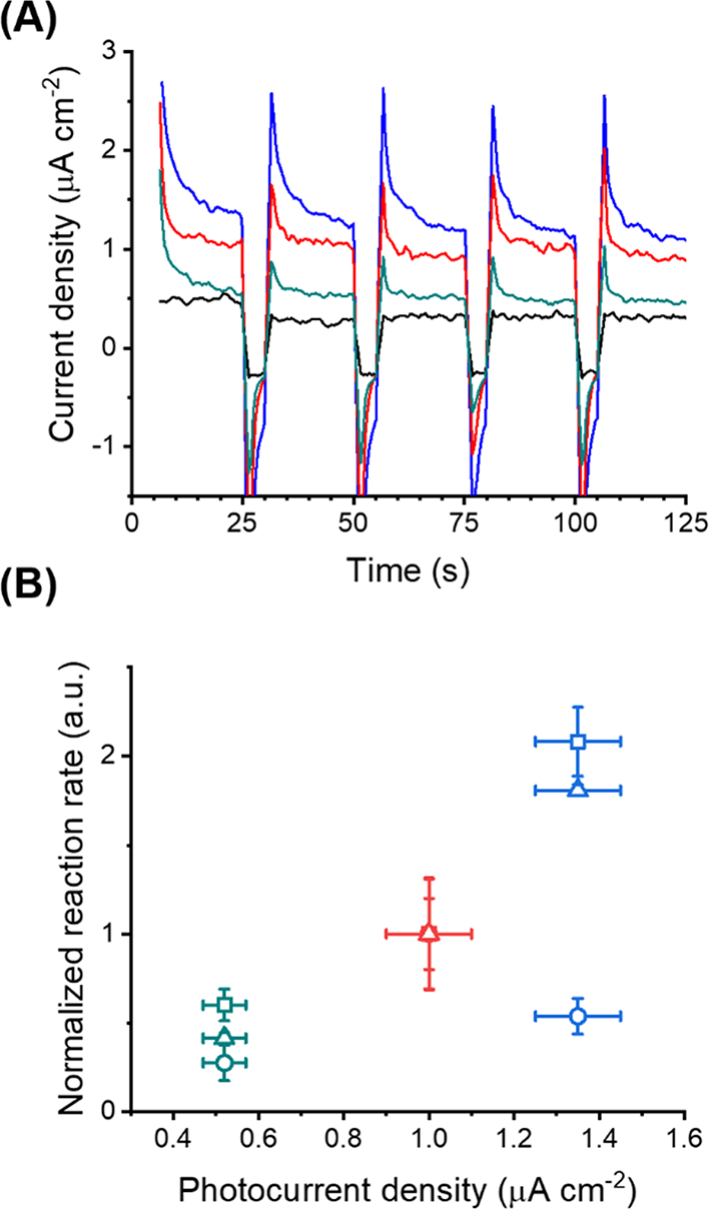
(A) Photocurrent measured with a PET/ITO electrode (black) covered with AuNSs (red), AuNRs (blue), and AuNTs (cyan). (B) Reaction rate for the photooxidation of rhodamine B (square), oxidation of phenol red (triangle), and reduction of 4-nitrophenol (circle) measured in the presence of NP producing different photocurrents, AuNSs (red), AuNRs (blue), and AuNTs (cyan).

Figure 5B shows that the photooxidation of rhodamine B and phenol red was faster in the presence of AuNRs than in the presence of AuNSs, which was faster than in the presence of AuNTs. However, the photoreduction of 4-NP was the fastest in the presence of AuNSs. The photocatalytic activity observed resulted from different factors, mainly the absorption of light resulting in the formation of hot electrons and the interaction between the reagents and the surface of the NPs. To qualitatively address the formation of hot electrons by light absorption, a constant concentration of AuNPs (2 mL of a suspension of 0.2 mg·mL^–1^) was immobilized on a 0.25 cm^2^ poly(ethylene terephthalate) film covered with indium tin oxide. The resulting electrodes were used in a three-electrode system to measure the photocurrent generated by their exposure to the white light used in the catalytic experiments (Figure 6). The results show that the current density generated by the AuNRs was larger than the current densities produced by the AuNSs and AuNTs (Figure 6A). The spectral overlap between the emission of the source (Figure S5) and the extinction of the NPs is another factor influencing the performance of the photocatalytic NPs. Interestingly, the AuNRs were the NPs with the poorest spectral overlap between the extinction of the NPs (Figure 3) and the emission of the white light used (Figure S5).

Consequently, while the generation of hot electrons by the NPs can explain the reactivity trend observed between the AuNSs, AuNRs, and AuNTs for the oxidation of phenol red and rhodamine B, the behavior observed for the conversion of 4-NP to 4-AP could not solely be attributed to the efficiency of hot electron formation. To understand the mechanism of the photocatalytic reactions, the reactivity of the different crystalline facets of gold and the AuNP composition also need to be taken into account. In the case of AuNSs, the particles present mostly (111) and (100) facets,^59^ while AuNTs mostly expose (111) planes,^60^ whereas in the AuNRs, the long cylindrical wall is built with (110) planes and spherical caps with (111) and (100) facets.^59^ The first step in the photooxidation reaction of phenol red and rhodamine B is the production of superoxide, such as O_2_^–^, from dissolved oxygen, and the formation of superoxide occurs preferentially at the (110) facet.^61^ In the case of the reduction of 4-NP, the first step of the reaction is the concomitant binding of 4-NP and borohydride ion to the surface of gold,^62^ which is favored on (100) facets. Consequently, the AuNSs, exposing a larger fraction of (100) facets, catalyzed the reduction of 4-NP more efficiently than the AuNTs and AuNRs.

**Figure 7:**
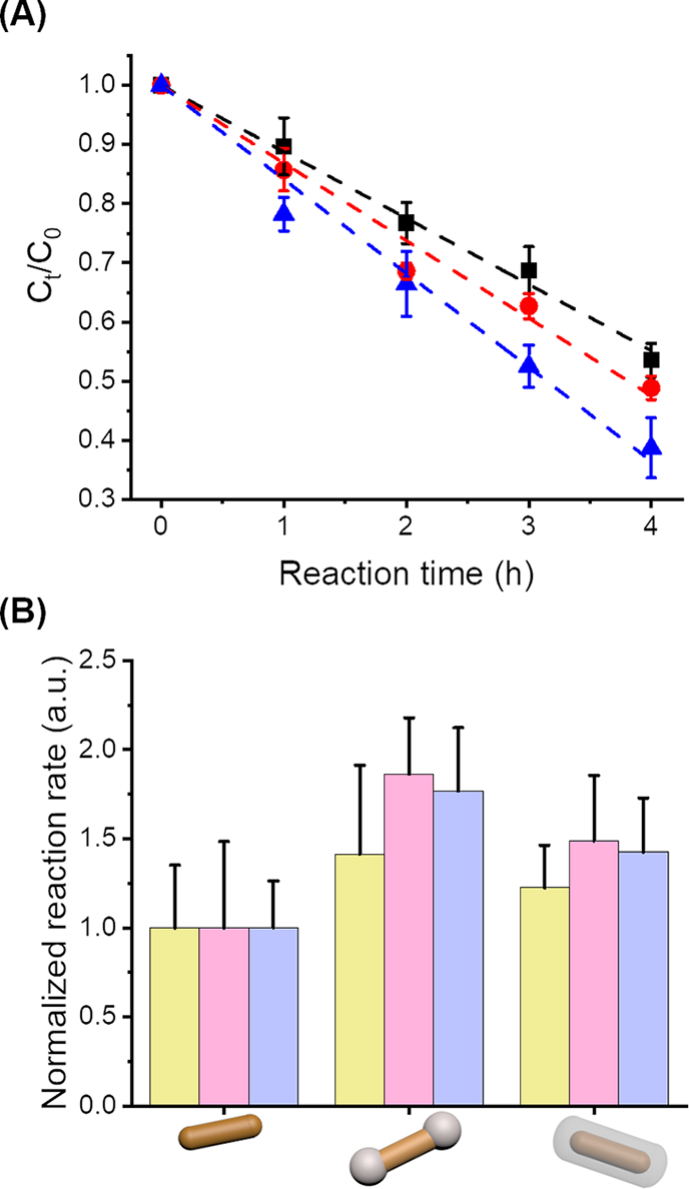
(A) Variation of the concentration of rhodamine B during photodegradation catalyzed with AuNR (square, black), AuNR@F-CeO_2_ (circle, red), and AuNR@T-CeO_2_ (triangle, blue). (B) Normalized reaction rate for the catalytical reaction of 4-nitrophenol to 4-aminophenol (yellow), of phenol red to bromophenol blue (blue), and the degradation of rhodamine B (pink) in the presence of AuNR@CeO_2_ with different CeO_2_ coverages.

The three benchmark photocatalytic reactions were also studied with different binary architectures (naked NPs, fully covered NPs, or partially covered NPs). The presence of CeO_2_ domains only had a marginal effect on the photocurrent measured during the irradiation of the three-electrode system (Figure S5). Nevertheless, the results show that the photocatalytic activity of the AuNPs increased with the addition of CeO_2_. However, the addition of a complete layer of CeO_2_ did not improve the performance of the photocatalyst in comparison to the partially covered AuNPs; either the same catalytic activity or a decrease in catalytic activity was observed for the fully covered AuNPs (Figure 7A). The catalytic activity of partially covered AuNP@CeO_2_ was higher than that of fully covered AuNP@CeO_2_, which was higher than the naked AuNPs. The results suggest that the access of the reacting molecules to the gold substrate influences the reaction rate, and the full coverage of the AuNPs with a layer of CeO_2_ impeded the diffusion of the reactive species to the reaction sites. However, the CeO_2_ layer was mesoporous due to the presence of CTAB during the synthesis of the ceria domains,^63^ and molecules can diffuse through the CeO_2_ patches to reach the gold surface. Consequently, the addition of CeO_2_ was limiting but not preventing the mass transport between the environment and the gold surface.

Furthermore, the gold surface is not the only potential locus of reaction. Under light irradiation, the AuNPs used as the template for the binary AuNP@CeO_2_ produced a pair of hot electrons and hot holes. Typically, those hot charge carriers can move to the gold surface to react with any molecules present there. In the case of binary NPs, the Au part was still responsible for the light absorption leading to the generation of hot charge carriers. However, while those hot charge carriers can still move to the gold surface, there is a second mechanism at play. The Schottky barrier at the Au/CeO_2_ interface favors the injections of the hot electrons from the AuNP to the conduction band of CeO_2_ (Figure 1), separating the electron–hole pair and effectively increasing the catalytic activity of the binary nanostructures.^23^ The electrons transferred to the CeO_2_ domains were available to perform photoreaction, and the CeO_2_ domains behaved as an electron transfer medium.

### Nanofibrous Hybrid Membranes

**Figure 8:**
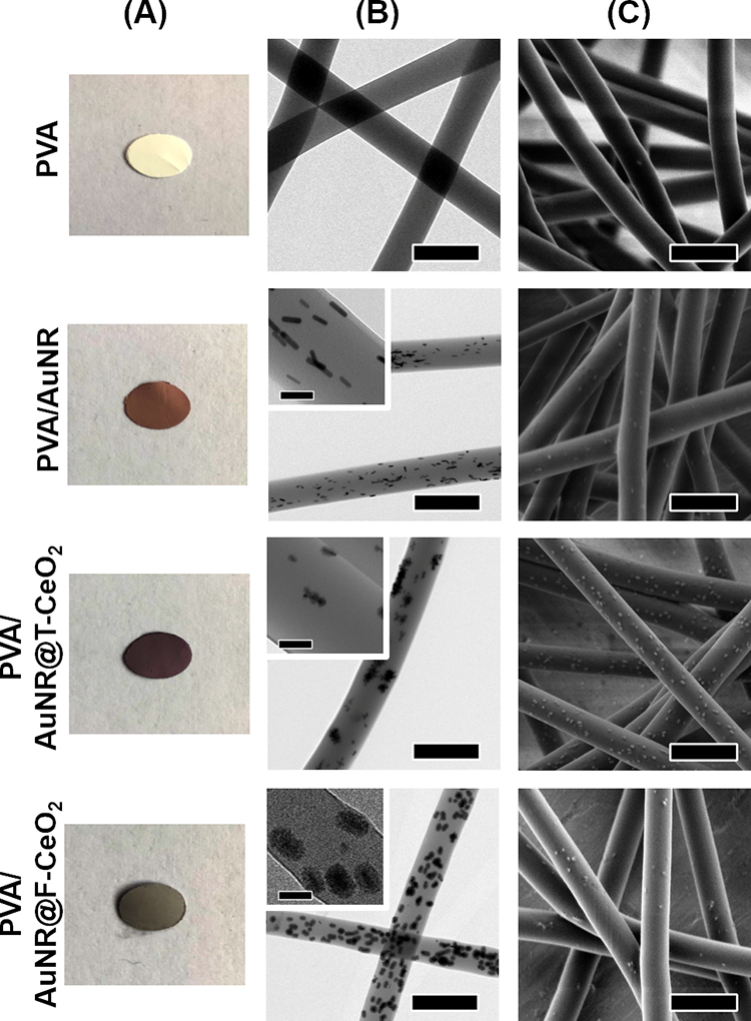
Electrospun hybrid membrane. (A) Images of the membrane coupons, (B) TEM images of the fibers (scale bars of 250 nm, 50 nm in the insets), and (C) SEM images of the fibers (scale bars of 250 nm) made with PVA or mixtures of PVA/AuNR, PVA/T-AuNR@CeO_2_, and PVA/F-AuNR@CeO_2_.

All AuNP@CeO_2_ photocatalysts prepared were highly efficient to catalyze the model reactions. However, their isolation from the reaction system was challenging. To prepare more convenient photocatalytic functional materials, the AuNR@CeO_2_ building blocks were immobilized in membranes made of poly(vinyl alcohol) (PVA) nanofibers (Figure 8). AuNR and AuNR@CeO_2_ were used as the model catalysts to prepare the catalytic membranes. The fibers were prepared by the colloidal electrospinning of a mixture of AuNR@CeO_2_ in suspension in a concentrated aqueous solution of PVA. All the hybrid nanofibrous membranes were prepared with the same loading of gold atoms (0.2 mg of Au/mg of PVA). The nanofibers were cross-linked (Figure S2) to prevent the dissolution of the membrane after immersion in water and efficiently entrap the NPs in the fibers, even after long immersions (Figure S7).

The AuNR@CeO_2_ NPs were distributed throughout the PVA fibers (Figure 8). The NPs with a more extensive coverage of CeO_2_ displayed some mild aggregation in the fibers, which was absent in the parent suspension used for electrospinning. Additionally, the NRs were oriented along the fiber axis direction. The alignment of NRs in PVA nanofibers can be attributed to the strong shear forces generated on the NR/PVA suspension during the electrospinning process. During electrospinning, the suspension was sheared by the flow driven by the difference of potentials applied, and the NRs were oriented along the direction of elongated polymer flow.^64,65^

The choice of a cross-linked PVA network as a matrix for the embedding of AuNR@CeO_2_ was made to allow access to the catalytic sites of the AuNPs for the molecules dissolved in water. Pure cross-linked PVA fibers swelled after immersion in water, and water-soluble reactants can penetrate the PVA fibers and react with the embedded NRs. Because of the combined permanent immobilization of the AuNP@CeO_2_ and access to the photocatalytic sites for small dissolved molecules, PVA/AuNR or AuNR@CeO_2_ nanofibrous membranes are promising hybrid materials for long-term use in aqueous environments.

**Figure 9:**
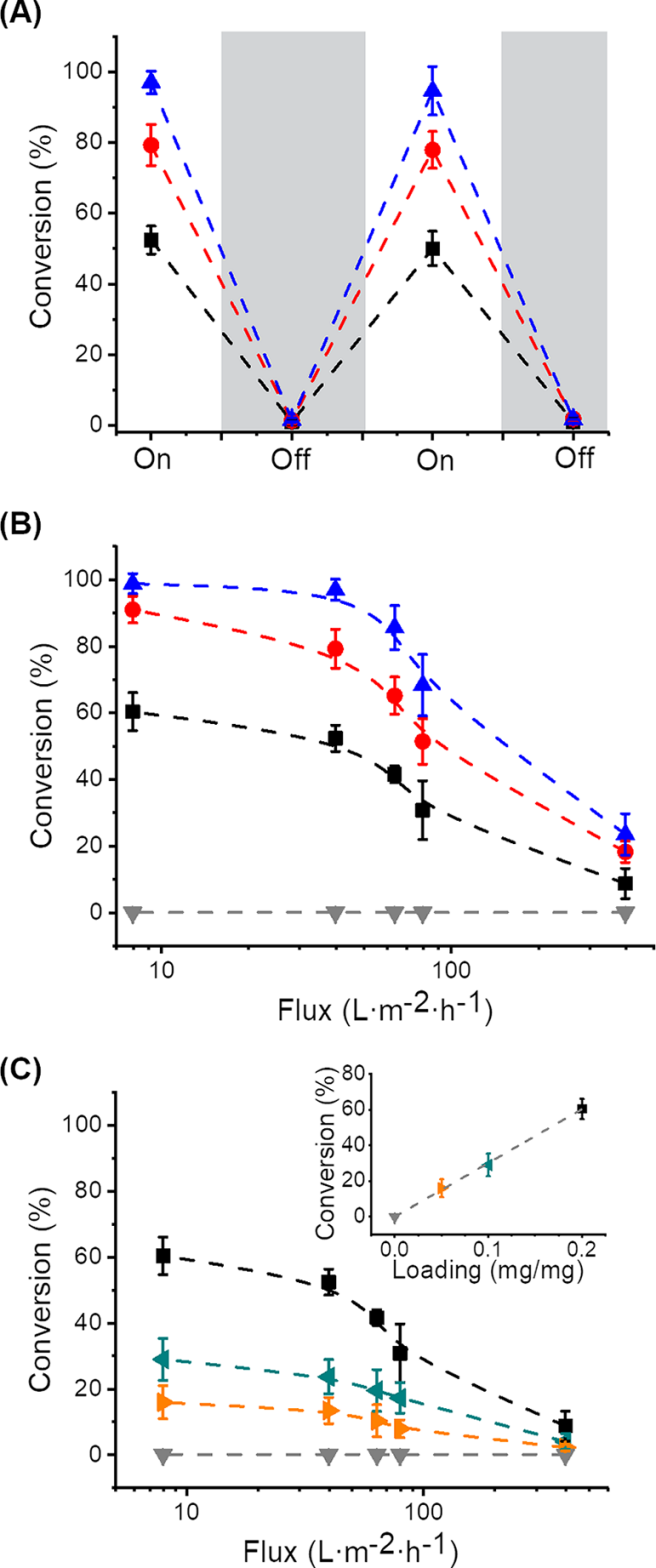
(A) Photodegradation of rhodamine B while switching the light irradiation on and off in a flow-through device and (B) degradation of rhodamine B using membranes prepared with AuNP@CeO_2_ with different structures (no AuNR (upside down triangle, gray), AuNRs (square, black), AuNR@F-CeO_2_ (circle, red), and AuNR@T-CeO_2_ (triangle, blue)); all the membranes were prepared with 0.2 mg of Au/mg of PVA. (C) Degradation of rhodamine B using membranes with variable loadings of AuNRs (0 mg of Au/mg of PVA (upside down triangle, gray), 0.05 mg of Au/mg of PVA (right-pointed triangle, orange), 0.1 mg of Au/mg of PVA (left-pointed triangle, cyan), and 0.2 mg of Au/mg of PVA (square, black).

The activity of the photocatalytic membranes was studied by their performances in the photodegradation of rhodamine B. PDMS devices were prepared to embed the nanofibrous membrane in a flow-through reactor (Figure 2). The reactor was fed a solution of rhodamine B, and the degradation of rhodamine B was monitored at different flow rates (Figure 9). The degradation of rhodamine B can be switched on and off by shining white light on the devices (Figure 9A). Similarly to free AuNR@CeO_2_ (Figure 7), the catalytic activity of the AuNR@CeO_2_ embedded in the PVA membranes was influenced by the type of CeO_2_ coverage. At a given flow rate, the membranes embedding AuNR@T-CeO_2_ displayed a higher photodegradation rate than the membranes containing AuNR@F-CeO_2_, which were more efficient than the membranes containing the naked AuNRs. Using a flow-through device, the rhodamine B conversion was no longer controlled by the reaction time but rather by the flux of rhodamine B solution through the membrane. During the experiments, the effective reaction time varied from ca. 0.06 to 3 s. When the flux of the solution was too fast, the residence time of the molecules in the photocatalytic area was not long enough to allow for a complete conversion of rhodamine B. Furthermore, the activity of the membranes can also be influenced by the loading of NPs in the fibers. As the concentration of NPs in the fiber increased, the rhodamine conversion observed at a given flow rate increased (Figure 9C). Interestingly, the fluidic cell can be used over a long period of time to perform the reaction without any significant decrease in the observed conversion (Figure S8).

## Conclusions

In summary, we prepared efficient photocatalytic membranes containing binary nanostructures made of gold and cerium oxide. Using the selective over-growth of crystalline cerium oxide on gold nanotemplates, we formulated a library of photocatalytic nanoparticles. Gold nanospheres, gold nanorods, and gold nanotriangles were used as templates for the growth of the cerium oxide domains, and the resulting binary nanoparticles catalyzed both reduction and oxidation photoreactions. The gold nanoparticles covered with patches of cerium oxide outperformed the fully covered and naked nanoparticles in terms of catalytic efficiency for every reaction tested. Conversely, the catalytic activity observed with the different gold templates was influenced by the reaction studied due to the different mechanisms involving the adsorption of specific chemical species on specific crystallographic facets. Finally, the most efficient binary nanostructures were successfully embedded in membranes by colloidal electrospinning. The photocatalytic activity was preserved in the hybrid nanofibrous membranes. The resulting functional membranes were a convenient and scalable manner to use the nanoparticles in model water remediation experiments. Owing to the combination of the rational design of photocatalytic nanoparticles and their efficient processing through electrospinning yielding highly efficient functional hybrid membranes, this type of hybrid nanofibrous material can find application in the fabrication of continuous photoreactors.

## Supporting Information Available

Detailed procedure for the synthesis of the binary nanoparticles and additional characterization of the nanoparticles and the hybrid nanofibrous membranes (PDF)
