# Supplementary material for: Nanofibrous Photocatalytic Membranes Based on Tailored Anisotropic Gold/Ceria Nanoparticles
Source: ACS Appl Mater Interfaces. 2021 Jul 30;13(31):37578–88. doi: 10.1021/acsami.1c11954 (PMC8365598; doi:10.1021/acsami.1c11954)
Supplement: Supplementary file 1 — am1c11954_si_001.pdf [file am1c11954_si_001.pdf]

Supporting Information

Nanofibrous Photocatalytic Membranes Based on Tailored Anisotropic Gold/Ceria Nanoparticles

Yinzhou Guo,[1] Héloïse Thérien-Aubin[1,2,*]

[1] Max Planck Institute for Polymer Research, Mainz, 55128, Germany

[2] Department of Chemistry, Memorial University of Newfoundland, St. John's, Newfoundland and Labrador, A1B 3X7, Canada

* Email: htherienaubin@mun.ca

Experimental section

Synthesis of the AuNP functionalized with CeO_2 domains (AuNP@CeO_2)

The following library of nanoparticle was synthesized:

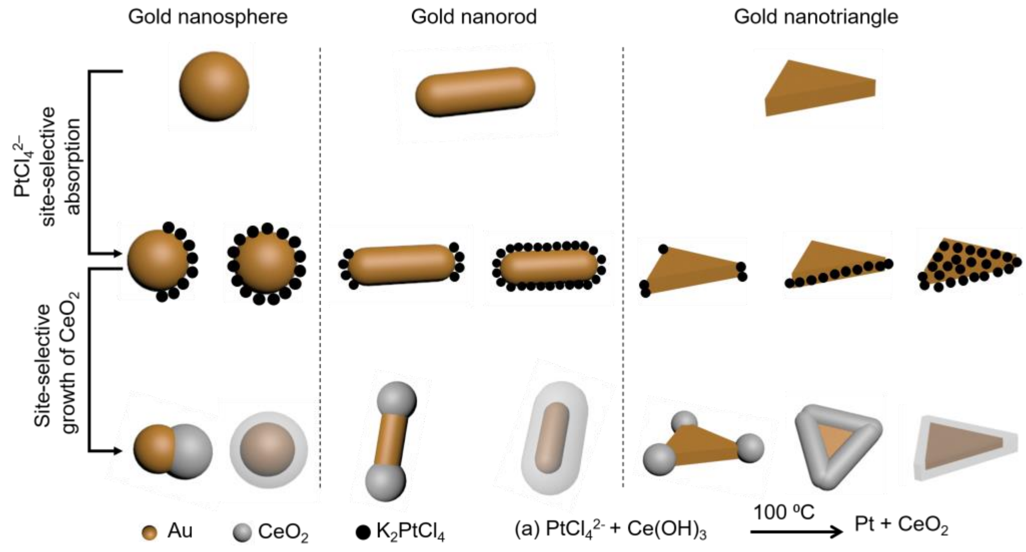

Figure S1. Schematic of synthesis different AuNP@CeO2 binary nanoparticles

Synthesis of the AuNS functionalized with CeO_2 domains (AuNS@CeO_2)

Typically, 1 ml of the as-prepared AuNS suspension was centrifuged (20,000 g, 30 min) and redispersed with 0.5 mL of CTAB solution (0.1 mM). Then, different amounts of K_2PtCl_4 solution (0.1 mM) were added to the AuNS suspension under gentle shaking. To get fully covered AuNSs (AuNS@F-CeO_2), 50 µL of platinum salt solution was added, and for the partially covered AuNSs (AuNS@J-CeO_2), 20 µL of the platinum salt solution was added. The resultant suspension was kept at room temperature for 30 min to allow for the adsorption of $PtCl_4^{2-}$ on the AuNSs. Then, a freshly prepared $Ce(AC)_3$ solution (10 mM) was added, either 200 µL for the synthesis of AuNS@F-CeO_2 or 100 µl for the AuNS@J-CeO_2. Then, water was added (250 µL for AuNS@F-CeO_2 or 380 µL for AuNS@J-CeO_2) to the suspension under gentle shaking. Finally, the resultant suspension was placed in an oven set at 100 °C for 1 h to produce the AuNS@CeO_2 binary nanoparticles. The unreacted salts were removed by two centrifugation cycles (15,000 g, 20 min, 27 °C), followed by redispersion in water.

Synthesis of AuNR functionalized with CeO_2 domains (AuNR@CeO_2)

Typically, 1 ml of the as-prepared AuNR suspension was centrifuged (12,000 g, 20 min) and redispersed with 0.5 mL of CTAB solution (0.1 mM). Then, different amounts of K_2PtCl_4 solution (0.1 mM) were added to the AuNR suspension under gentle shaking. To get fully covered AuNRs (AuNR@F-CeO_2), 50 µL of platinum salt solution was added, and for the tips covered AuNRs (AuNR@T-CeO_2), 10 µL of the platinum salt solution was added. The resultant suspension was kept at room temperature for 30 min. Then, a freshly prepared $Ce(AC)_3$ solution (10 mM) was added, either 200 µL for the synthesis of AuNR@F-CeO_2 or 30 µl for the AuNR@T-CeO_2. Then, water was added (250 µL for AuNR@F-CeO_2 or 410 µL for AuNR@T-CeO_2) to the suspension under gentle shaking. Finally, the resultant suspension was placed in an oven set at 100 °C for 1 h to produce the AuNR@CeO_2 binary nanoparticles. The unreacted salts were removed by two centrifugation cycles (10,000 g, 20 min, 27 °C), followed by redispersion in water.

Synthesis of AuNT functionalized with CeO_2 domains (AuNT@CeO_2)

Typically, 1 ml of the as-prepared AuNT suspension was centrifuged (1,000 g, 20 min) and redispersed with 0.5 mL of CTAB solution (0.1 mM). Then, different amounts of K_2PtCl_4 solution (0.1 mM) were added to the AuNT suspension under gentle shaking. To get fully covered AuNTs (AuNT@F-CeO_2), 100 µL of platinum salt solution was added, and for the edge covered AuNTs (AuNT@E-CeO_2), 30 µL of the platinum salt solution was added. The resultant suspension was kept at room temperature for 30 min. Then, a freshly prepared $Ce(AC)_3$ solution (10 mM) was added, either 250 µL for the synthesis of AuNT@F-CeO_2 or 50 µl for the AuNT@E-CeO_2. Then, water was added (150 µL for AuNT@F-CeO_2 or 420 µL for AuNR@E-CeO_2) to the suspension under gentle shaking. Finally, the resultant suspension was placed in an oven set at 100 °C for 1 h to produce the AuNT@CeO_2 binary nanoparticles. The unreacted salts were removed by two centrifugation cycles (800 g, 20 min, 27 °C), followed by redispersion in water.

Crosslinking of the nanofibers

After electrospinning, the poly(vinyl alcohol) in the electrospun mats was reacted with glutaraldehyde (a dialdehyde) in an acidic atmosphere to form cyclic acetal bonds efficiently crosslinking the PVA matrix in the nanofibers (Figure S2).

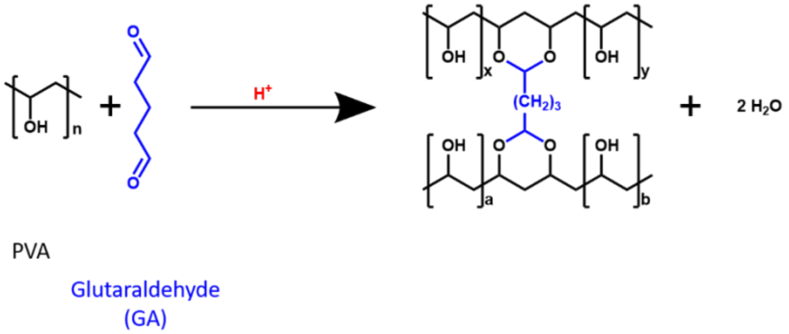

Figure S2. Crosslinking of the poly(vinyl alcohol) by glutaraldehyde to form crosslinked poly(vinyl alcohol) nanofibers.

Characterization

Powder X-ray diffraction (XRD)

Solid-state analysis of the naked AuNPs and AuNP@CeO_2 was conducted on a STOE Stadi P diffractometer using Ag Kα_1 radiation. The sample was prepared on a perfluoropolyether film with Fomblin Y. The data were analyzed with the EVA software package from Bruker. All diffraction peaks were assigned either to the cubic phase of CeO_2 (JCPDS #34-394) or to the cubic phase of Au (JCPDS #89-3697).

UV-Vis absorption spectroscopy

The absorbance of the suspensions and solutions was acquired on a Agilent Cary 60 UV-vis spectrometer between 300 - 1000 nm.

Thermogravimetric Analysis (TGA)

The measurements were conducted using a Mettler-Toledo TGA/SDTA-851 thermobalance (50–700 °C, heating rate of 5 °C·min^{-1}, nitrogen atmosphere; the initial sample weight was ca. 10 mg).

SEM and TEM imaging

The morphology of the NPs was imaged using SU8000 scanning electron microscope (Hitachi, Japan) and a JEM1400 transmission electron microscope (JEOL, Japan). TEM samples were prepared from casting a 5 μL of NPs suspension on a 300 mesh copper grid covered with carbon film and dried quickly by removing the solvent at room temperature with a filter paper.

Additional results.

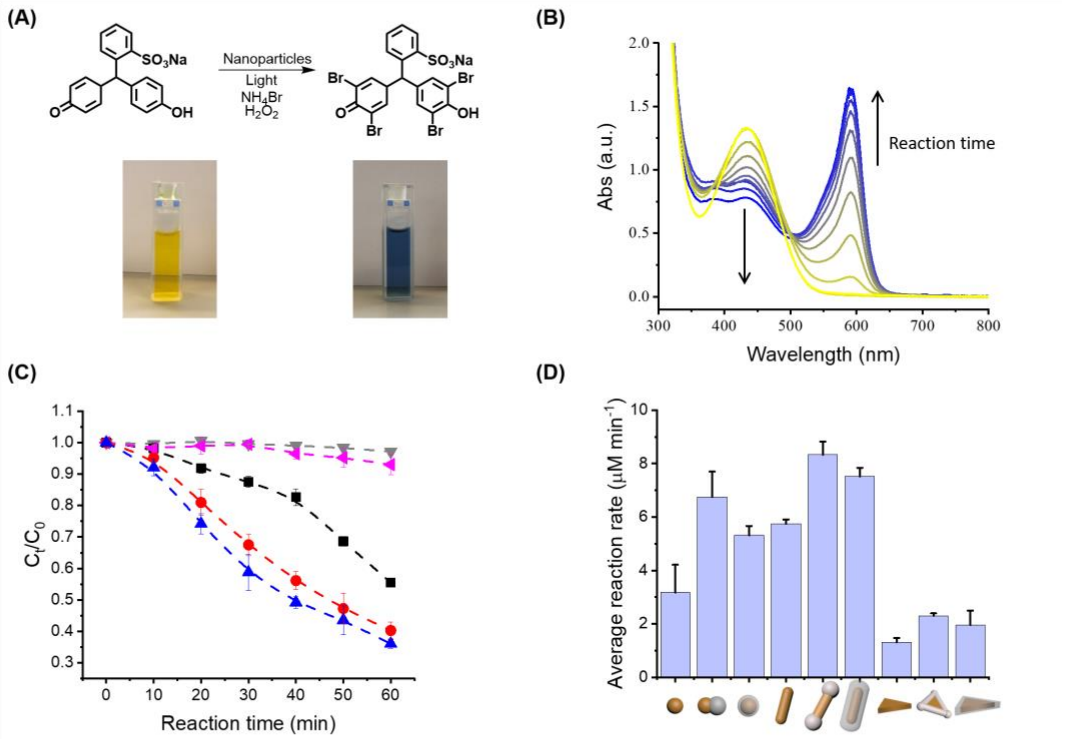

Figure S3. Catalytic activity of the nanoparticles in suspension during the photooxidation of phenol red. (A) Conversion of phenol red to bromophenol blue. (B) UV–vis spectra of the catalytic conversion of phenol red to bromophenol blue. (C) Variation of the concentration of phenol red during the reaction in the presence of AuNRs, or AuNR@CeO_2, under different conditions. (▼) light irradiation, without AuNRs; (◀) with AuNRs, without irradiation; (■) with AuNRs, under irradiation; (▲) with AuNRs@T-CeO_2, under irradiation; and (●) with AuNRs@F-CeO_2, under irradiation. (D) Reaction rate of the photoconversion of phenol red in bromophenol blue in the presence of the different AuNP@CeO_2.

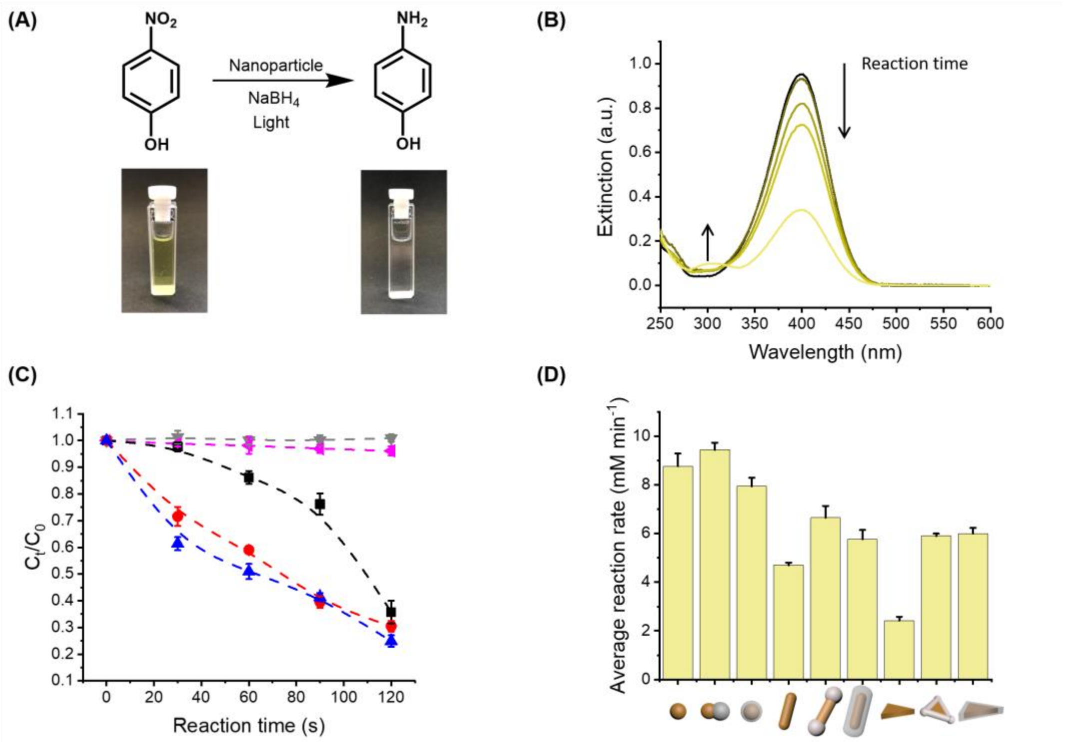

Figure S4. Catalytic activity of the nanoparticles in suspension during the photoreduction of 4-nitrophenol. (A) Conversion of 4-nitrophenol to 4-aminophenol. (B) UV–vis spectra of the catalytic conversion of 4-nitrophenol. (C) Variation of the concentration of 4-nitrophenol during the reaction in the presence of AuNRs, or AuNR@CeO_2, under different conditions. (▼) light irradiation, without AuNRs; (◄) with AuNRs, without irradiation; (■) with AuNRs, under irradiation; (▲) with AuNRs@T-CeO_2, under irradiation; and (●) with AuNRs@F-CeO_2, under irradiation. (D) Reaction rate of the photoconversion of 4-nitrophenol in the presence of the different AuNP@CeO_2.

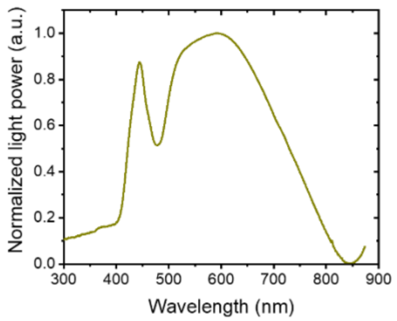

Figure S5. Emission of the white light used in the photocatalytic experiments.

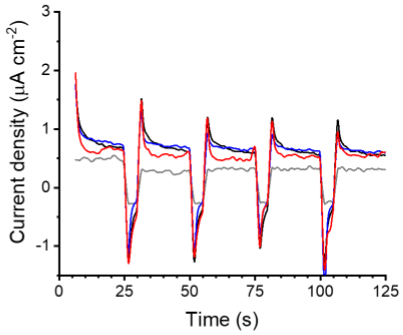

Figure S6. Photocurrent measured for PET/ITO electrode (grey) covered with AuNRs (black), AuNR@T-CeO_2 (blue) and AuNR@F-CeO_2 (red).

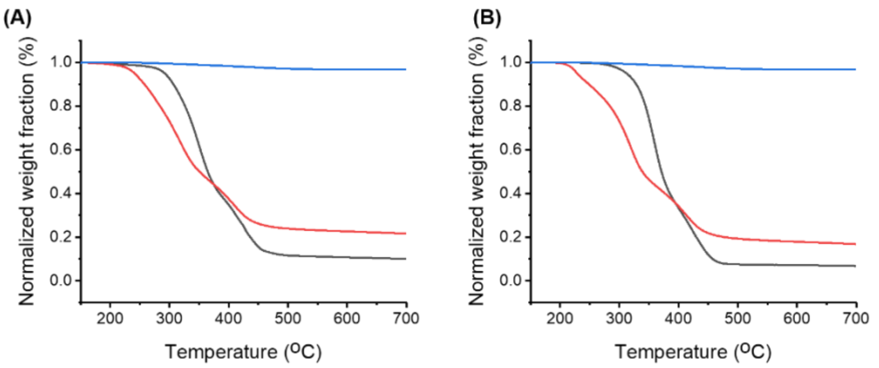

Figure S7. Thermogravimetric analysis of pure PVA membrane (black), PVA/AuNR membrane (red) and pure AuNR (blue) before (A) and after (B) immersion in water for 2 weeks.

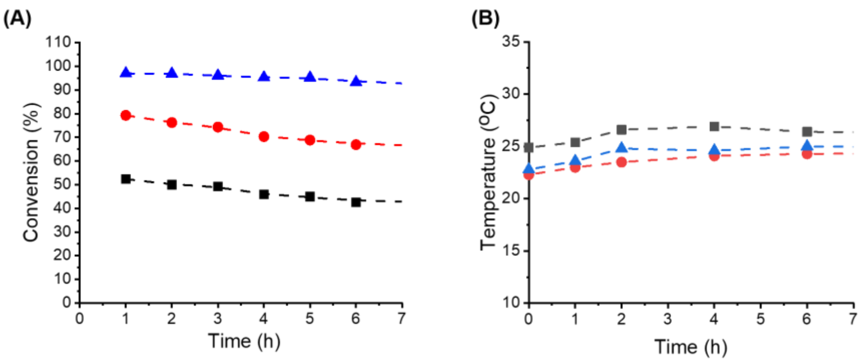

Figure S8. Stability of the photocatalytic membranes during long-term operation. (A) Variation of the efficiency of the catalytic membranes for the degradation of rhodamine B (B) Variation of the temperature at the surface of the membrane during operation. For membranes prepared with AuNRs (black), AuNR@F-CeO_2 (red) and AuNR@T-CeO_2 (blue).
